# Quantitative T2 mapping monitoring the maturation of engineered elastic cartilage in a rabbit model

**DOI:** 10.1186/s12880-023-00985-9

**Published:** 2023-03-06

**Authors:** Guojun Yang, Xue Li, Weiwei Zhang, Nier Wu, Haifeng Chen, Xia Liu, Haiyue Jiang

**Affiliations:** 1 Auricular Plastic and Reconstructive Surgery Center, Plastic Surgery Hospital, Chinese Academy of Medical Sciences, Peking Union Medical College, 33 Badachu Road, Shijingshan District, 100144 Beijing, People’s Republic of China; 2 Institute of Basic Medical Sciences, Chinese Academy of Medical Sciences, Peking Union Medical College, 5 Dongdan Santiao, Dongcheng District, 100005 Beijing, People’s Republic of China; 3 Department of Biomedical Engineering, College of Engineering, Peking University, 5 Yiheyuan Road, Haidian District, 100871 Beijing, People’s Republic of China; 4 Research Center, Plastic Surgery Hospital, Chinese Academy of Medical Sciences, Peking Union Medical College, 33 Badachu Road, Shijingshan District, 100144 Beijing, People’s Republic of China

**Keywords:** Tissue engineering, Elastic cartilage, Non-invasive assessment, MRI, T2 mapping

## Abstract

**Background:**

Cartilage tissue engineering provides a promising approach to reconstruct craniofacial defects, and a noninvasive method is needed to assess its effectiveness. Although magnetic resonance imaging (MRI) has been used to evaluate articular cartilage in vivo, few studies focused on its feasibility in monitoring engineered elastic cartilage (EC).

**Methods:**

Auricular cartilage, silk fibroin (SF) scaffold, and EC consisting of rabbit auricular chondrocytes and SF scaffold were transplanted subcutaneously into the rabbit back. In eight weeks after transplantation, grafts were imaged by MRI using PROSET, PDW VISTA SPAIR, 3D T2 VISTA, 2D MIXED T2 Multislice, and SAG TE multiecho sequences, followed by histological examination and biochemical analysis. Statistical analyses were performed to identify the association between T2 values and biochemical indicator values of EC.

**Results:**

In vivo imaging shows that 2D MIXED T2 Multislice sequence (T2 mapping) clearly distinguished the native cartilage, engineered cartilage and fibrous tissue. T2 values showed high correlations with cartilage-specific biochemical parameters at different time points, especially the elastic cartilage specific protein elastin (ELN, r= -0.939, P < 0.001).

**Conclusion:**

Quantitative T2 mapping can effectively detect the in vivo maturity of engineered elastic cartilage after subcutaneously transplantation. This study would promote the clinical application of MRI T2 mapping in monitoring engineered elastic cartilage in the repair of craniofacial defects.

**Supplementary Information:**

The online version contains supplementary material available at 10.1186/s12880-023-00985-9.

## Introduction

Elastic cartilage exists in the external ear, the eustachian tube and the larynx, which presents a dense network of elastin fibers. Cartilage has a poor capacity for healing, and current strategy to reconstruct elastic cartilage deformities relies on autologous cartilage transplantation. However, this operation causes donor site morbidity as well as requiring extensive surgical expertise [1, 2].

The tissue engineering technique provides a promising option for the restoration of damaged tissue [3, 4]. Our group has developed tissue-engineered cartilage and conducted clinical research for auricle reconstruction of five microtia patients and achieved a satisfactory aesthetical outcome for 2.5 years follow-up in the first case [5]. At present, histopathological examination is still recognized as the clinical gold standard for evaluating the engineered cartilage maturation. However, histopathological examination is invasive and cannot monitor the in vivo outcome of tissue-engineered cartilage in real-time. To popularize clinical application of tissue-engineered cartilage, the issue of noninvasive detection needs to be solved.

Magnetic resonance imaging (MRI) provides high resolution in vivo images with excellent soft tissue contrast without invasive operations. Recent study indicated the effectiveness of MRI in quantifying water contents and extracellular components of cartilage in the osteoarthritis model [6]. T2 mapping is one of the main MRI techniques to detect early degenerative changes in cartilage. T1 rho mapping may have an advantage in differentiating grades I and II cartilage degeneration on OARSI (Osteoarthritis Research Society International) histological grading system [7], and T2 Mapping has been used as a new method for quantitative biochemical assessment of cartilage damage in rheumatoid arthritis [8]. Considering the components of elastic cartilage are different from the hyaline cartilage of joints, we need to confirm whether this technique effectively indicates the in vivo maturation of engineered elastic cartilage.

**Fig. 1:**
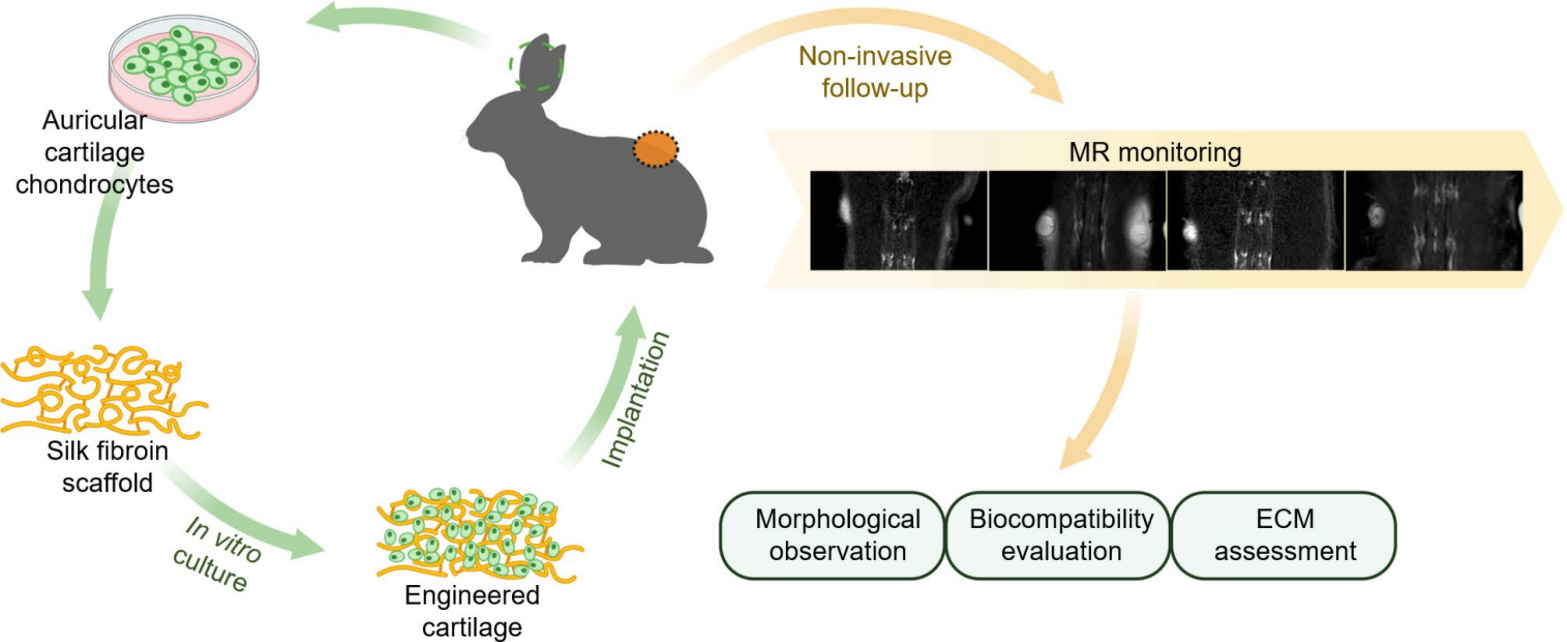
Schematic illustration of the study design and timeline

This study aims to verify the efficiency of T2 mapping in monitoring in vivo maturation of engineered elastic cartilage in immunocompetent animals. For this purpose, the rabbit auricular chondrocytes were isolated, passaged and seeded on the silk fibroin scaffold to construct the engineered elastic cartilage. The constructs were cultured in vitro for four weeks and autologously transplanted into the back of rabbits subcutaneously. At different time points after transplantation, the grafts were analyzed by MRI to quantify the T2 values. Simultaneously, the constructs were harvested for histological and biochemical analysis and analyzed for correlation with T2 values (Fig. 1).

## Materials and methods

### Chondrocytes harvest, isolation, and expansion

Auricular cartilage was harvested from the right ear of New Zealand white rabbits (aged within 2 months). Chondrocytes were isolated via enzymatic digestion. Cells were cultured to 80% confluence on a T-flask (Sigma-Aldrich, USA) with Dulbecco’s modified Eagle’s Medium (DMEM) (HyClone, USA) containing 10% fetal bovine serum (FBS) (Gibco, USA) and 1% antibiotic mixture (10,000 units penicillin, 10 mg streptomycin, and 25 ug amphotericin B per ml, Sigma-Aldrich, USA) at 37 ◦C in a humidified atmosphere of 95% air and 5% CO_2_ for 5 to 7 days.

### In vitro construction of tissue-engineered elastic cartilage

The silk fibroin (SF) scaffolds were offered by Dr. Haifeng Chen’s lab from the Department of Biomedical Engineering, Peking University, which was obtained by the freeze-drying method and used according to the literature ^24^. Briefly, sterilized SF scaffolds (10 mm × 10 mm × 2 mm) were rinsed for 12 h with sterile PBS, vacuum-dried, and then incubated in DMEM at 37 ◦C for 24 h. The chondrocyte suspension (4 × 10^7^ cells/ml) was seeded onto the SF scaffold (200 ul per scaffold) and incubated at 37 *°* C for 3 h. Afterwards, 2 ml of cell culture medium containing 10% FBS and 1% antibiotic mixture was added. The cell-scaffold constructs were cultured for four weeks in vitro at 37 *°*C and 5% CO_2_.

### Scanning electron microscope (SEM) examination

The cell-free scaffold and engineered cartilage cultured for one week in vitro were fixed in 4% glutaraldehyde for 12 h and then coated with gold after drying. A scanning electron microscopy (SEM; S-4800, Hitachi, Tokyo, Japan) was used to examine the samples at 1 kV.

### Animal experiments

Four healthy matured Zealand white rabbits (experimental animal center, Plastic Surgery Hospital, Chinese Academy of Medical Sciences, Peking Union Medical College, Beijing, China) which weighed on average 3 kg (3.0 ± 0.8 kg) were used. The rabbits were transplanted with cell-free silk fibroin scaffolds (SF), engineered elastic cartilage (EC), and autologous auricular cartilage (AC) on pre-designed back sites respectively.

Rabbits were anesthetized with 10% ketamine (0.5 mg/kg, i.m.) and 2% xylazine (0.5 mg/kg, i.m.). Three group grafts were implanted subcutaneously on the back of the rabbits. After transplantation, the rabbits were anesthetized at different time points, followed by magnetic resonance (MR) scanning and construct harvesting surgery.

All animal care and experimental procedures were approved by the Institutional Animal Care and Use Committees at Peking Union Medical College in keeping with the Institutional Animal Care and Use Committee guidelines.

### Magnetic resonance imaging (MRI) and quantitative of T2 value

MR scanning was performed using a 3.0 T MR system (Philips Ingenia, Netherlands) with a 32-channel head coil. The protocol consisted of a PDW VISTA SPAIR sequence, a PROSET sequence, a 3D T2 VISTA sequence, a SAG UTE TORSO sequence, and a 2D MIXED T2 Multislices clear sequence (T2 mapping). Table 1 summarized the imaging parameters. MRI data acquisition and reconstruction were performed using the Philips software system (Philips Medical System). All MRI measurements were carried out at room temperature. The MRI morphological images were processed by a radiologist (Le He, Biomedical Imaging Research Center of Tsinghua University) under the supervision of a second radiologist (Hua Guo, Biomedical Imaging Research Center of Tsinghua University) both with a specialty in musculoskeletal MRI, using the Philips Medical System. Each segmentation was reviewed by the second radiologist and changes were made following a consensus process. Three regions of interest (ROIs) were delineated: the auricular cartilage (AC) ROI, engineered cartilage (EC) ROI, and silk fibroin scaffold (SF) ROI. Each ROI was segmented in the 2D MIXED-T2 Multislice sequence. The absence of animal motion in multi-echo sequence was visually checked and manual registration of the ROI was provided if necessary. The ROIs were then imported into the Philips IntelliSpace Portal workstation (Philips Medical System) and automatically superimposed on the T2 maps.

**Table 1:** Imaging parameters for MRI protocols

Sequence	Preparation parameters	Slice Thickness (mm)
PDWVISTA SPAIR	TR = 8.5 ms. TE = 4.6 ms.	1.2
PROSET	TR = 2300 ms. TE = 31.5 ms.	1.2
SAG UTE TORSO	TR = 5.4 ms. TE = 0.1 ms.	1.2
3D T2 VISTA	TR = 2300 ms. TE = 200 ms.	1.1
2D MIXED T2 Multislices clear	TR = 2000 ms. TE = 20, 40, 60, 80, and 100 ms.	1.2

The mean T2 values were determined as the average of T2 values of each construct in a series of continuous T2 maps.

### Histology examination

After MR scanning, the constructs were excised from the body to perform the histological staining. Constructs were harvested and carefully dissected from the surrounding tissue. Samples for histology staining were embedded in paraffin and sectioned. Sections were stained with hematoxylin and eosin (H&E) staining; cartilage extracellular matrix (ECM) formation was assayed with Safranin O Fast green and Masson Trichrome (MT) staining; Organization of collagen fibers in constructs was observed by picrosirius red staining under a polarized microscope (DM2500; Leica, Wetzlar, Germany) [9].

### Biochemical analysis

The concentration of sulfated glycosaminoglycan (GAG), elastin (ELN), collagen type 1 (COL 1), collagen type 2 (COL 2), and total hydroxyproline (HYP) content, in the auricular cartilage and engineered cartilage was assayed using commercially available ELISA kits (Jianglai Biotechnology, China) according to manual instruction. Absorbance was measured at 450 nm with a Microplate reader (PE, USA).

### Quantitative reverse transcription polymerase chain reaction (qRT-PCR)

mRNA was extracted from samples by TRIzol (Invitrogen, USA). The cDNA was reverse transcribed using HiFiScript cDNA (Kangweishiji, China). The reaction comprised an initial denaturation for 95 ℃ for 3 min, 40 cycles with denaturing at 95 ℃ for 3 s, and annealing and extension at 60 ℃ for 22 s in each cycle, performed using a StepOne Real-Time PCR System (Applied Biosystems, USA). Rabbit *β-actin* was used as an endogenous control for the study. The primers used were shown in Table 2. The relative gene expression profiles of samples were normalized to the corresponding *β-actin* and analyzed using the 2^−∆∆CT^ approach. Three replicates were made per sample.

**Table 2:** Primers used in quantitative real-time PCR (qRT-PCR)

Primer	Forward (5’ − 3’)	Reverse (5’ − 3’)
*β-actin*	TTGTCCCCCAACTTGAGATGTA	GCACTTTTATTGAACTGGTCTCGT
*COL 2*	AGGATGGCTGCACGAAACA	CCCTATGTCCACCCCGAAT
*COL 1*	TGTCGATGGCTGCACGAAA	GGGCCAACGTCCACATAGAA
*ACAN*	TGCACAGTCCCTCAGCAATG	GTGGTTCTGCTTGTCCAGGAA

### Statistical analysis

SPSS 20.0 (IBM Corporation, Armonk, NY, USA) was used for statistical analysis. Data were presented as mean ± SD (standard deviation of the mean) One-way analysis of variance (ANOVA) was employed with Bonferroni’s honestly significant difference post hoc test to investigate the differences in T2 values within groups and within in vivo time. Pearson’s correlation analysis was applied to assess the linear relationship between the T2 values and the biochemical data. P < 0.05 was considered statistically significant.

## Results

### Assessment of engineered constructs in vitro

**Fig. 2:**
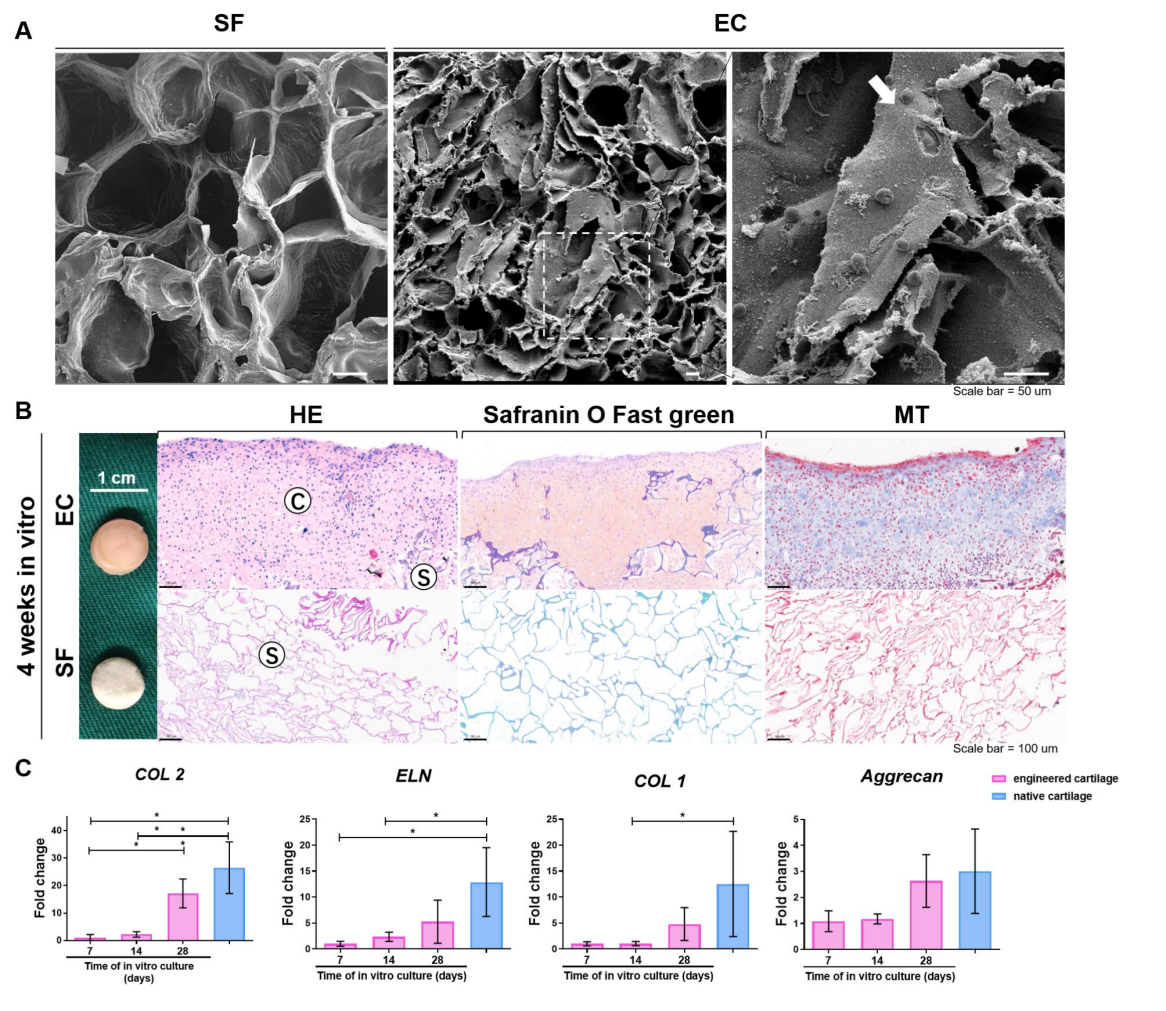
Characterization of constructs cultured in vitro. (A). Representative Scanning electron micrograph (SEM) images of cell free SF scaffold (SF) and chondrocyte-scaffold construct (EC) after 1week of culture in vitro. Scale bar = 50 μm. White arrow indicates the filopodia growing from the chondrocyte. (B). Representative macroscopic and histological images of constructs after 4 weeks in vitro culture. “C” and “S” refer to the cartilaginous and the scaffold region, respectively. Scale bar = 100 μm. (C). Collagen type II (*COL 2*), type I (*COL 1*) and *Aggrecan* mRNA expression analysis by quantitative Real-time PCR. All data were normalized to the corresponding *β-actin* value at each time point (2^-∆∆CT^) and further normalized to the mean value of the target gene in the constructs after 1-week in vitro culture. Data is expressed as mean fold change SD from at least three independent experiments. Statistical differences were calculated using ANOVA and Bonferroni’s test. * P < 0.05; ** P < 0.01. Abbreviations: EC: engineered cartilage, SF: silk fibroin scaffold, H&E: hematoxylin-eosin staining, MT: Masson’s Trichrome staining

SF scaffold exhibited a pore size of approximately 100–150 μm in diameter by SEM examination (Fig. 2A). In the EC group, the cells adhered to the scaffold with filopodia formation. After four weeks of culturing, the chondrocyte-scaffold constructs (EC) exhibited an ivory-like appearance. H&E staining showed that cartilage lacuna formed and the scaffold partly degraded during 4w in vitro culture. Safranin-O staining showed that cartilage-specific ECM such as glycosaminoglycan (GAG) was expressed (Fig. 2B).

qRT-PCR (Fig. 2C) showed that the expression of *COL 2*, *COL 1* and *Aggrecan* was increased in engineered cartilage during in vitro culturing, and showed no significant difference with the native auricular cartilage at 4w (all P > 0.05).

### In vivo histological and biochemical evaluation of the engineered cartilage

**Fig. 3:**
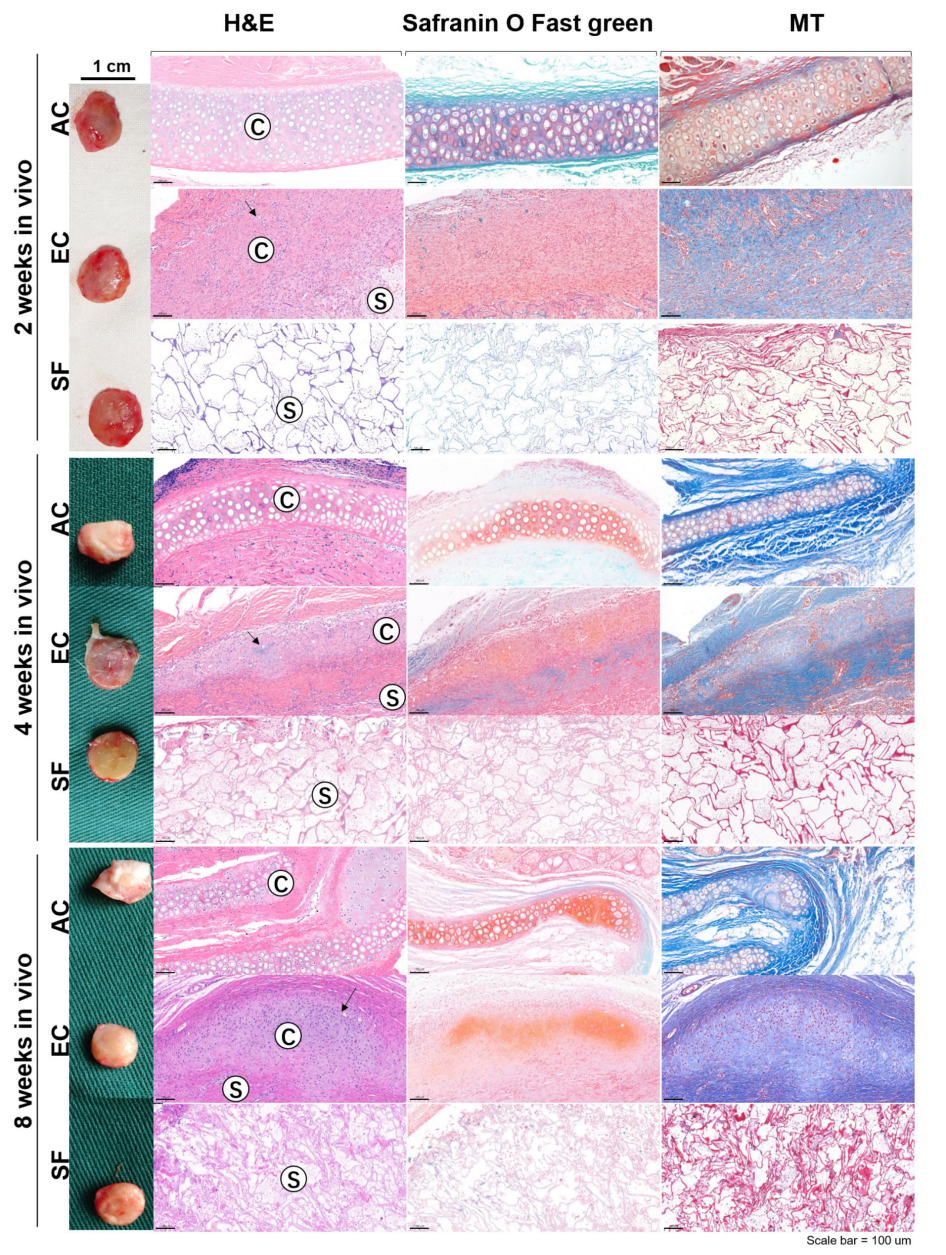
Macroscopic evaluation and histological staining of samples after in vivo implantation. Representative images of H&E, Safranin O Fast green and MT staining. “C” and “S” refer to the cartilaginous and the scaffold region, respectively. Scale bar = 100 *u*m. Abbreviations: AC: autologous auricular cartilage grafted in the subcutaneous layer. EC: engineered cartilage. SF: silk fibroin scaffold. MT: Masson’s Trichrome staining

The constructs were autologously implanted in the dorsal back of the donor rabbit subcutaneously after 4w of culture. At 2w, 4w and 8w post-implantation, constructs were harvested and submitted for histological examination. In the AC group, the cartilage grafts had no significant changes macroscopically for 8w, while H&E staining showed a partial absorption of cartilage tissue at 8w in vivo, showing as the cartilage tissue layer became thinner (Fig. 3). In the EC group, the accumulations of GAG as well as collagen was observed at 8w in vivo. In the SF group, the scaffold was wrapped with connective tissue and gradually degenerated, together with leukocytes and fibroblasts infiltration over time (Fig. 3).

**Fig. 4:**
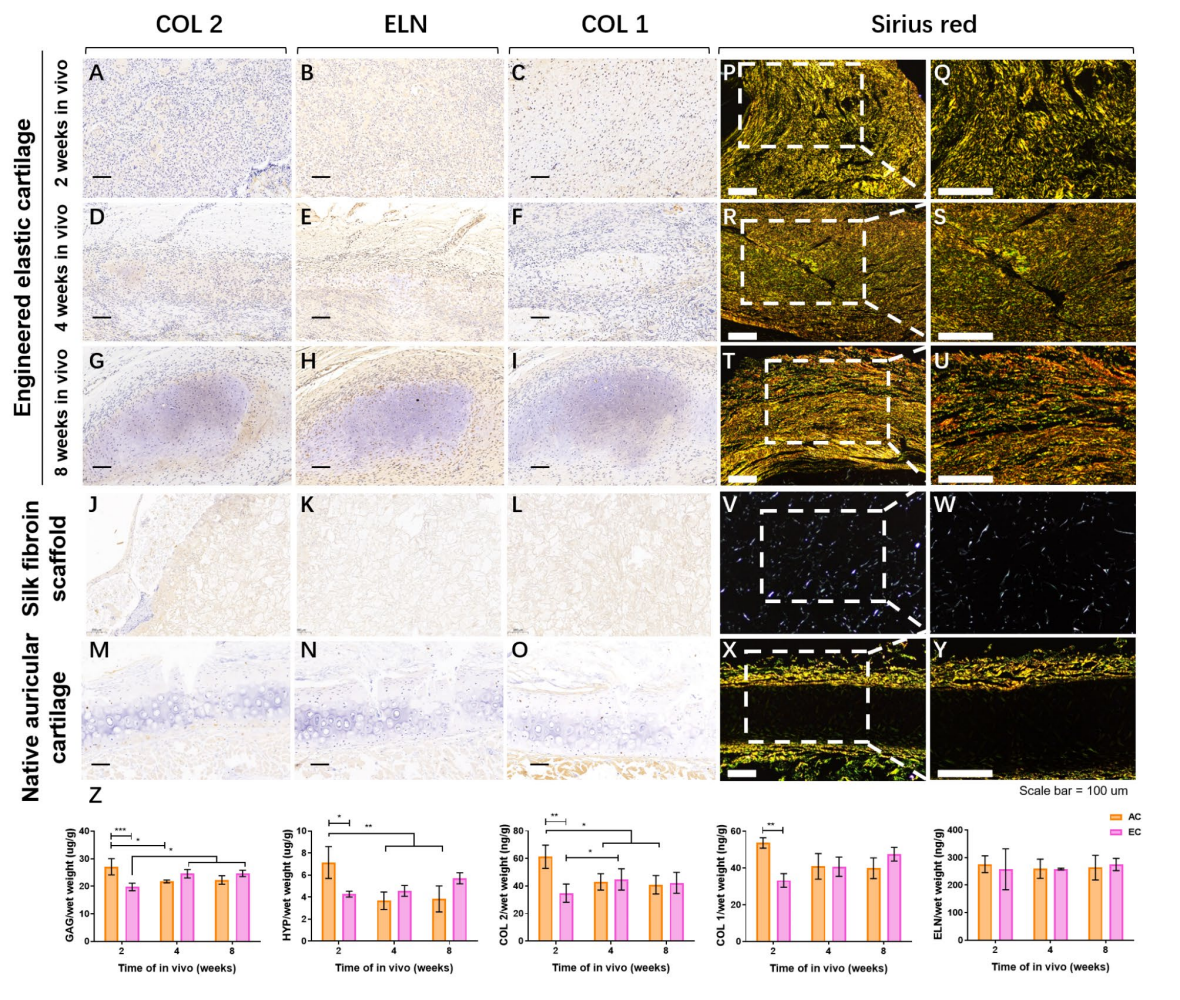
Histological examination and protein evaluation of engineered cartilage after in vivo implantation. (A-O). Representative images of immunostaining for collagen type II (COL 2), elastin (ELN) and collagen type I (COL 1) in engineered cartilage, native ear cartilage and silk fibroin scaffold; (P-Y). picrosirius red staining examination under a polarized microscope; (Z). Quantification of glycosaminoglycan (GAG), total hydroxyproline (HYP), COL2, COL 1 and elastin (ELN), content of EC and AC constructs overtime. Scale bar = 100 μm. Data is expressed as the mean *±* SD (n *≥* 3). * P < 0.05; ** P < 0.01. Abbreviations: AC: autologous auricular cartilage grafted in the subcutaneous layer. EC: engineered cartilage

Immunohistochemical staining of ECM-featured proteins in the engineered cartilage after in vivo transplantation (Fig. 4A-O) showed positive for COL 2, ELN and COL 1, and their expression increased over time. Picrosirius red staining was performed to observe the collagen organization within the engineered cartilage (Fig. 4P-Y). Using polarized light microscopy, the type I collagen, which appear as brilliant (strongly birefringent) red-yellow fibers and the greenish collagen III fibers (weakly birefringent) were clearly visualized in the perichondrium of the rabbit ear cartilage. In the EC group, the brilliant red-yellow fibers and green fibers increased over time, suggesting the maturation and deposition of collagen fibers.

Cartilage specific protein quantification test further confirmed the histological results. The autologous auricular cartilage grafts gradually degraded after implantation, showing that the content of GAG, HYP, and COL 1 decreased over time. Besides, the GAG, HYP, COL 1, and COL 2 contents of the AC grafts were significantly higher than those of the EC constructs at 2w in vivo, while there was no significant difference between the two groups at 4w and 8w. In addition, the ELN content was relatively stable in both EC and AC groups after implantation (Fig. 4Z).

### In vivo MRI evaluation of the engineered cartilage

**Fig. 5:**
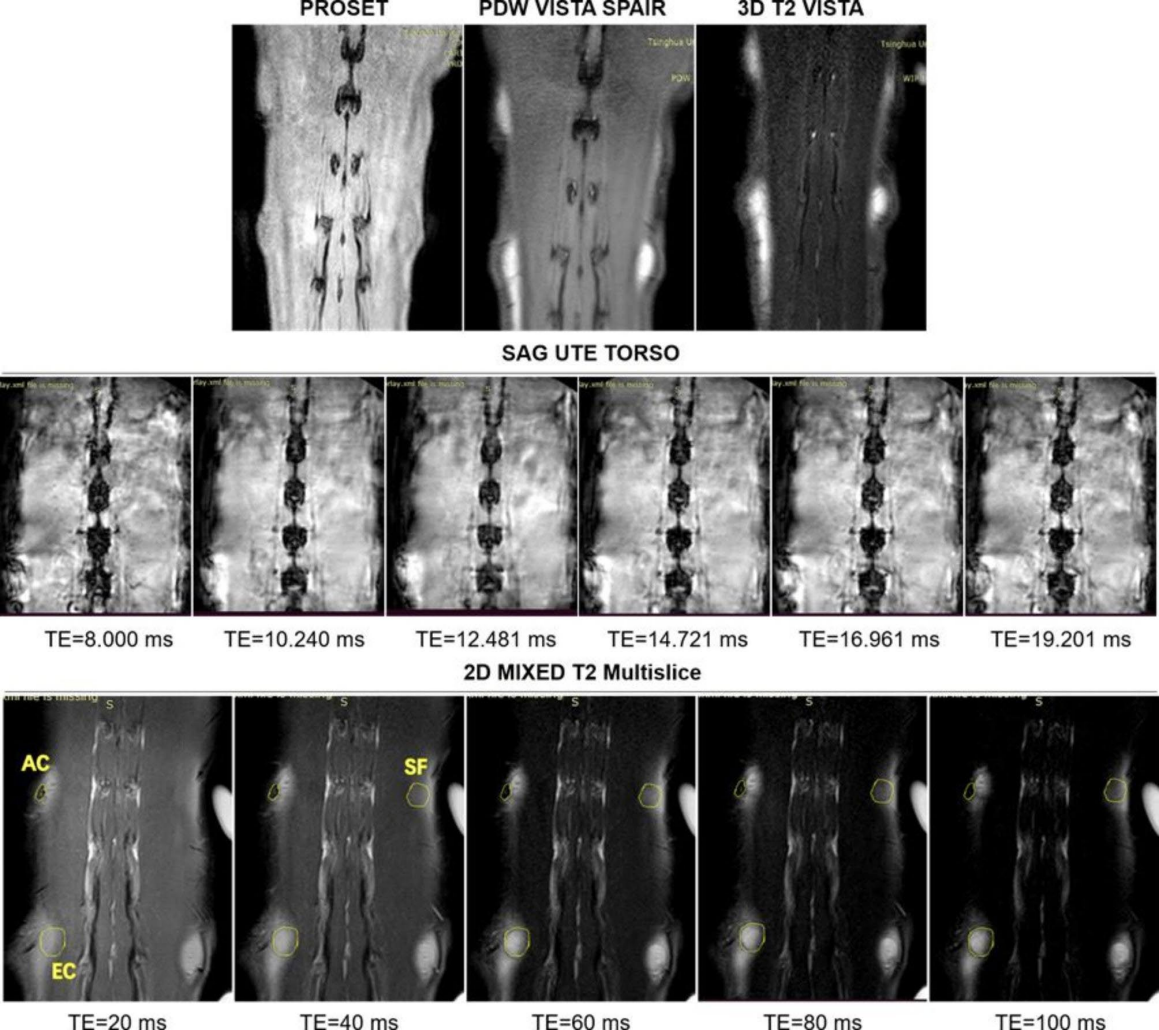
MRI evaluation of samples at 1 week after implantation using different sequences. Abbreviations: AC: autologous auricular cartilage grafted in the subcutaneous layer. EC: engineered cartilage. SF: silk fibroin scaffold

**Fig. 6:**
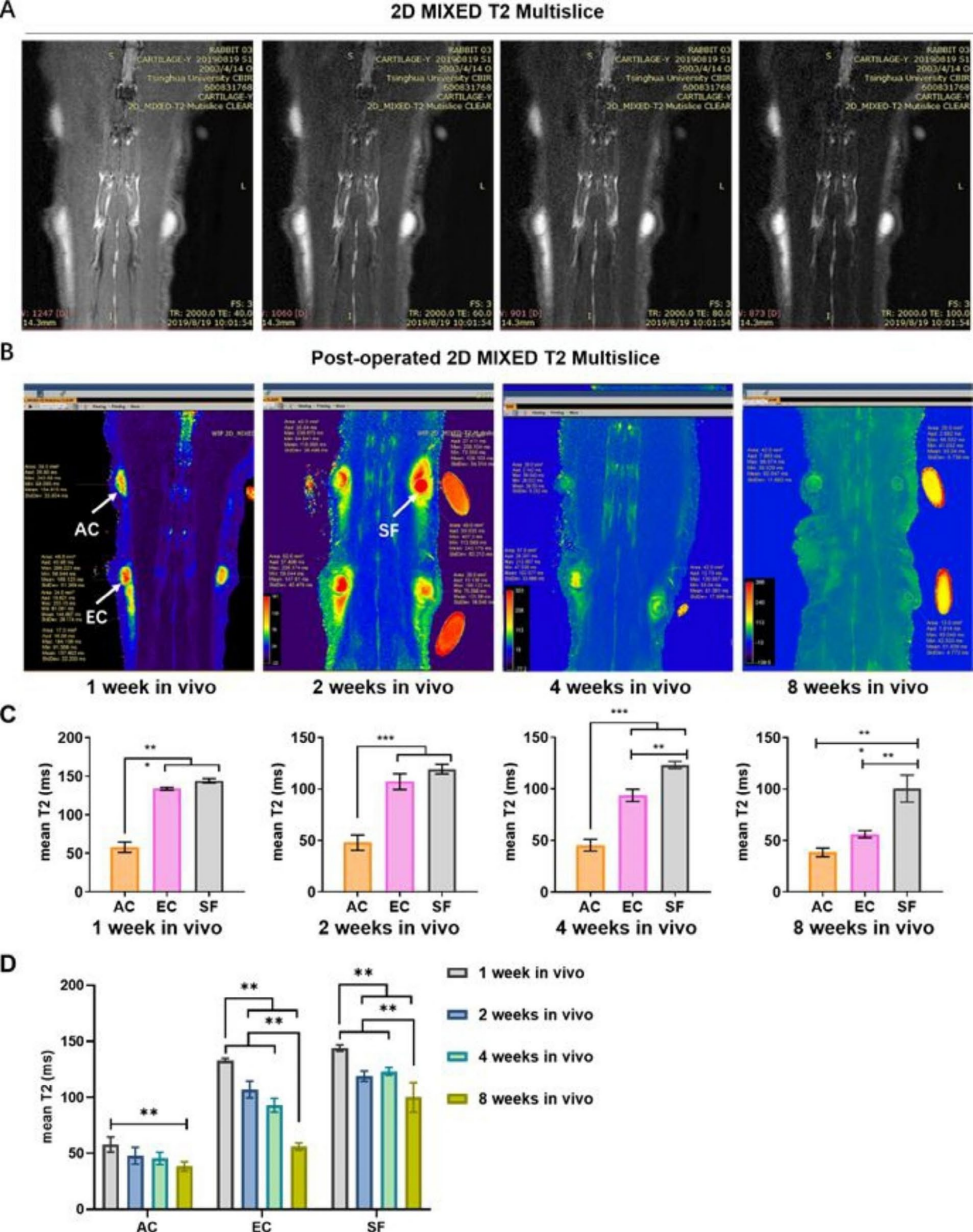
MRI T2 mapping evaluation of samples after implantation at different time point. (A). Representative images of samples in the AC (upper left), SF (lower left) and EC (right) groups at 1w after implantation. (B). Measurement of T2 values on post-operated 2D MIXED-T2 Multislice images overtime. (C). Mean full thickness T2 values (milliseconds) of the samples at different time point. (D). Mean full thickness T2 values (milliseconds) in each group at the indicated follow-up times. Data is expressed as the mean ± SD. Statistical differences were calculated using ANOVA and Bonferroni’s test. * P < 0.05, ** P < 0.01. # indicates statistical significance (p < 0.01) between the marked group and all other groups. Abbreviations: AC: autologous auricular cartilage grafted in the subcutaneous layer. EC: engineered cartilage. SF: silk fibroin scaffold

The PROSET, PDW VISTA SPAIR, 3D T2 VISTA, 2D MIXED T2 Multislice, and SAG TE multiecho sequences were performed to detect the grafts 1w after implantation. We found that the 2D MIXED T2 Multislice sequence provided higher quality images within a short time and was more sensitive to cartilaginous tissue compared with the other sequence (Fig. 5). Thus, we used 2D MIXED T2 Multislice to generate T2 mapping images in this study (Fig. 6A &B). The quantitative results (Fig. 6C & D) showed that the average T2 value of grafts in the AC group was significantly lower than that in the EC and SF groups from 1w to 4w after implantation (P < 0.001). It was also noted that the T2 changes in the three groups were asynchronous in vivo (Fig. 6D). In the AC group, the T2 values did not significantly change until 8w when they showed significantly lower values than at the beginning of implantation. In spite of the fact that the EC group had significantly higher T2 than the AC group at 1w in vivo, this level of T2 declined rapidly thereafter. At 8w in vivo, there was no significant difference in T2 values between AC and EC groups (P = 0.085). The average T2 value of the grafts in the SF group also decreased (144.1 ± 2.83 ms and 100.4 ± 13.13 ms in 1w and 8w in vivo, respectively), but it was still significantly higher than that in the EC and AC groups after 8w.

### Correlation analysis between the T2 value and biochemical indicators value

**Fig. 7:**
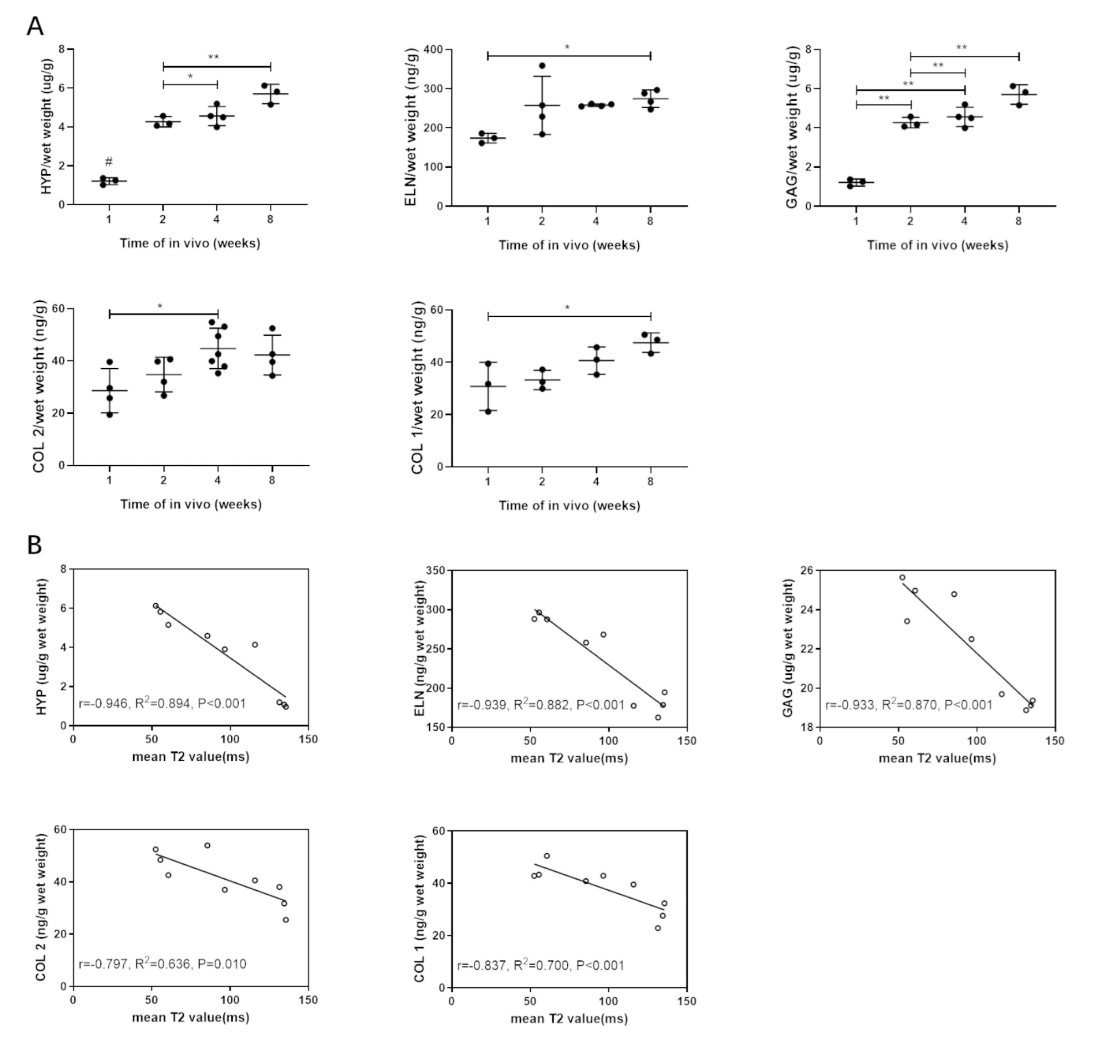
Biochemical properties and mean T2 values of EC constructs at different time point. (A). total hydroxyproline (HYP), elastin (ELN), Glycosaminoglycan (GAG), COL 2 and COL 1 content of the engineered cartilage overtime. Data is expressed as the mean ± SD (n *≥* 3). (B). Scatterplots for the relationship between the biochemical properties and T2 values of cartilage. Solid line represents the linear regression line. * P < 0.05; ** P < 0.01; P < 0.001. # indicates statistical significance (p < 0.01) between the marked group and all other groups

To further determine the engineered cartilage maturity, we quantified the ECM-featured proteins at 1w, 2w, 4w and 8w after implantation. The results showed that the contents of HYP, ELN, GAG, COL 2, and COL 1 gradually increased over a period of eight weeks in vivo (Fig. 7A). HYP content significantly increased from the second week in vivo, while GAG and Col 2 increased significantly from the fourth week, and ELN protein content increased significantly until the eighth week in vivo. Notably, T2 values were more sensitive to the in vivo maturation, showing significant differences at all four-time points.

To clarify the feasibility of using T2 values to indicate the biochemical index of the engineered cartilage, the Pearson correlation analysis between the T2 value and biochemical indicators value was done (Fig. 7B). We found that T2 significantly correlated with total collagen, ELN and GAG contents (collagen: r = -0.946, P < 0.001; ELN: r= -0.939, P < 0.001; GAG: r = -0.933, P < 0.001). Moderate correlations were found between T2 and specific COL 1 or COL 2 content (COL 1: r = -0.837, P = 0.005; COL 2: r = -0.797, P = 0.01).

## Discussion

In this study, elastic cartilage was constructed using auricular chondrocytes and cross-linked silk fibroin scaffolds (SFs). In an immunocompetent animal model (New Zealand rabbit), we showed the in vivo maturity process of the engineered elastic cartilage (EC) over a period of 8 weeks by histological and biochemical analysis. T2 mapping was used to characterize and quantify the MRI properties. By correlating T2 values with ECM contents, we confirmed that MRI T2 mapping could monitor the in vivo regeneration of SF-based engineered cartilage to some extent. To our knowledge, our study was the first to involve quantitative T2 mapping to monitor engineered elastic cartilage in immunocompetent animal models.

Tracing engineered cartilage characteristics at different growth stages by a noninvasive method is needed to assess the success of tissue regeneration techniques. MRI has been recognized as an effective method for non-invasive detection of articular cartilage [10, 11]. The ECM of cartilage mainly consists of water (60–85% of wet weight) and macromolecules such as collagen fibers and proteoglycan [12]. Specifically, T1 relaxation time was found to be highly correlated with the proteoglycan content, and water apparent diffusion coefficient (ADC) were found to reflect the total amount of collagen and proteoglycans [13, 14].

Considering that the components of hyaline cartilage of joints and elastic cartilage of the ear differ in ECM content, we need to confirm whether MRI can be used to assess the engineered elastic cartilage maturity. In our previous study, we used MRI spin-lattice relaxation time (T1) to indicate the degradation of the PCL-PGA scaffold, however, it was not enough to monitor the maturity of engineered cartilage yet [5]. T2 mapping is a newer MRI technique that can calculate a sequence of T2 times of a given tissue and display them voxel-vice on a parametric map. With T2 mapping, no contrast administration is needed, and three-dimensional imaging can be carried out [15, 16]. Fujihara et al. [14] demonstrated that T2 was highly sensitive to collagen content and tissue hydration in engineered cartilage implanted in nude rats. However, the regeneration process of engineered tissues in immunocompromised animals and immunocompetent ones is very different. We found that edema caused by acute inflammation would manifest as a high T2 value, so it was difficult to assess the constructs at the early-stage post-implantation. More specifically, after 4-week’s in vitro culturing, chondrocytes-SF scaffold constructs gradually formed engineered cartilage, confirmed by histological results and mRNA expression of cartilage-featured genes. However, shortly after implantation (2 weeks), the mean T2 value of the EC group was significantly higher than that of AC control, meanwhile the difference of T2 values between the EC and SF group was not significant. One possible explanation is the inflammatory response and the resulting in situ edema caused by the scaffold materials.

In this study, we used SF as the scaffold. Silk fibroin, a self-assembling structural protein in natural silkworm fibers, is broadly used as scaffolds in tissue engineering because of its excellent mechanical properties, biocompatibility, and inductive formation of crystalline β-sheet structure networks [17]. In cartilage tissue engineering, SF-based scaffold seeded with articular chondrocytes and mesenchymal stem cells (MSCs) showed satisfying ECM deposition [18, 19]. Meanwhile, studies showed that inflammatory response caused by natural polymers is significantly lower but still exists [20, 21]. We used gamma-ray irradiation to induce the intermolecular cross-linking of SF fibers, which increased the mechanical strength and reduced the biodegradation period of SF [22]. The results showed that the scaffold didn’t degrade completely at eight weeks in vivo and would still cause acute inflammation after being implanted in the body. Therefore, how to avoid the influence of early inflammatory edema caused by material implantation on MRI imaging is a problem that needs to be considered in the application of MRI detection.

Meanwhile, we also noted a decrease of T2 value in the SF and AC group, which can be explained by the infiltration of fibrous connective tissue in the SF group, and the cartilage tissue degradation in the AC group. Comparing the T2 values among AC, EC, and SF groups, we confirmed that MRI could indicate the difference between normal cartilage, engineered elastic cartilage, and fibrous tissue. Moreover, the T2 value showed a better correlation with GAG and HYP contents of tissue-engineered cartilage, which was consistent with other studies [14, 18, 23]. Especially, the T2 value showed a high coefficient of more than 0.88 with elastin content, suggesting the feasibility of MRI T2 mapping in evaluating engineered elastic cartilage. In contrast, the T2 value had little correlation with collagen content. Advanced techniques such as diffusion tensor MRI (DT-MRI) [24] and small-angle X-ray scattering (SAXS) have been demonstrated to possess great sensitivity in evaluating the distribution and orientation of the collagen fibrils [25]. These techniques should be considered in future studies to provide quantitative information of cartilage tissue growth at all stages.

## Conclusion

This study confirmed the feasibility of MRI T2 mapping in distinguishing the native cartilage, engineered cartilage and fibrous tissue, and can be further used to monitor the maturation process of engineered elastic cartilage in immunocompetent animals. We clarified the linear correlations between T2 relaxation times and cartilage-specific proteins, especially GAG and elastin contents in engineered elastic cartilage. Therefore, MRI T2 mapping is an applicable non-invasive technique to monitor the engineered cartilage in vivo in future craniofacial clinical applications.

## Electronic supplementary material

Below is the link to the electronic supplementary material.

Supplementary Material 1

## Data Availability

The datasets used and/or analysed during the current study available from the corresponding author on reasonable request.
