# Supplementary material for: Quantitative T2 mapping monitoring the maturation of engineered elastic cartilage in a rabbit model
Source: BMC Med Imaging. 2023 Mar 6;23:36. doi: 10.1186/s12880-023-00985-9 (PMC9987110; doi:10.1186/s12880-023-00985-9)
Supplement: Supplementary file 1 — Supplementary Material 1 [file 12880_2023_985_MOESM1_ESM.docx]

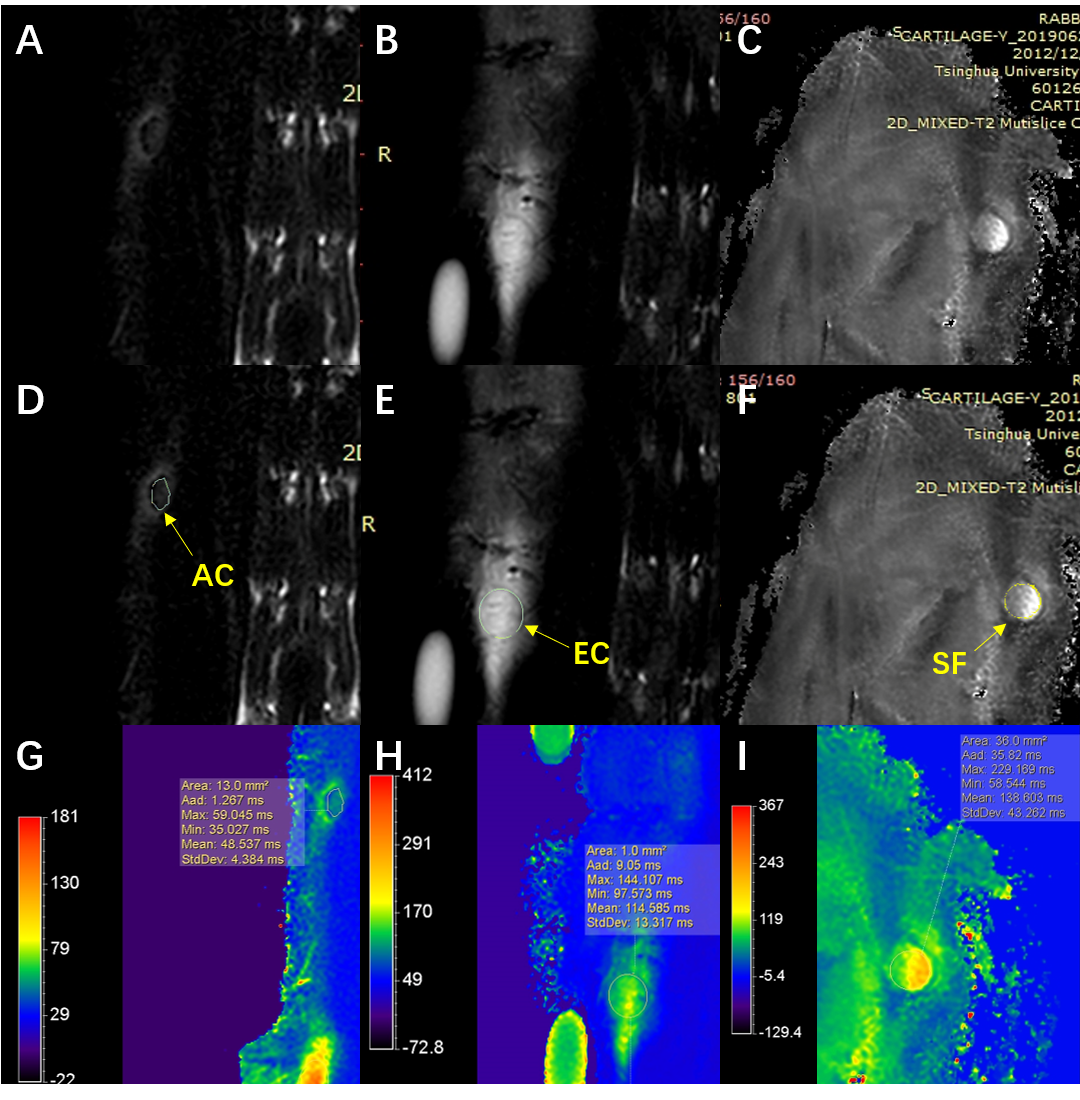
Supplementary material

Figure S1. Definition and transposition of the regions of interest (ROIs). (A-C). Representative images of auricular cartilage (AC), engineered cartilage (EC), and silk fibroin scaffold (SF) area on the 2D MIXED-T2 Multislice sequence performed in Philips Medical System. (D-F) Manual delineation of the samples. (G-I). The same ROIs transposed on the T2 map.
